# Depressive disorder, bipolar disorder, and associated factors among adults, in the Eastern part of Ethiopia

**DOI:** 10.1186/s12888-023-05466-5

**Published:** 2024-01-03

**Authors:** Tilahun Bete, Tadesse Misgana, Kabtamu Nigussie, Kemal Aliye, Tilahun Abdeta, Dawud Wedaje, Gari Hunduma, Abduselam Assefa, Dejene Tesfaye, Henock Asfaw, Abdulkarim Amano, Mandaras Tariku, Jerman Dereje, Tilahun Ali, Fethia Mohammed, Mekdes Demissie, Ahmed Mohammed, Nejiba Hayru, Birhanu Assefa, Tara Wilfong, Daniel Alemu

**Affiliations:** 1 Department of Psychiatry, School of Nursing and Midwifery, College of Health and Medical Sciences, Haramaya University, Harar, Ethiopia; 2 School of Medicine, College of Health and Medical Sciences, Haramaya University, Harar, Ethiopia; 3 School of Public Health, College of Health and Medical Sciences, Haramaya University, Harar, Ethiopia; 4 School of Nursing and Midwifery, College of Medicine and Health Sciences, Wollo University, Dessie, Ethiopia

**Keywords:** Depression, Bipolar disorder, Poverty, Unemployment, Social support, Substance abuse, Eastern part of Ethiopia

## Abstract

**Background:**

Depressive disorder is one of the severe and common mental illnesses in the general population. Bipolar disorder is a severe, persistent mental illness associated with significant morbidity and mortality. However, there is a paucity of data on the prevalence of depressive disorder, and bipolar disorder in our study area.

**Objective:**

This study aimed to assess the prevalence of depressive and bipolar disorders among adults in Kersa, Haramaya, and Harar Health and Demographic Surveillance Sites in Eastern Ethiopia.

**Methods:**

A community-based cross-sectional study was conducted among 1,416 participants. A multi-stage sampling was employed to select the participants. DSM-5 diagnostic criteria was used to assess depressive disorder and bipolar disorder. Data was collected using a standard questionnaire. Data were entered into Epi-Data 3.1 and analyzed using SPSS version 26. Both binary and multivariate logistic regression analyses were done. Those with a *p*-value < 0.05 in the final model were considered statistically significant.

**Results:**

The overall prevalence of depressive and bipolar disorders among our study participants was 6.7% (95% CI: 5.40, 8.20) and 2.1% (95% CI: (1.40, 3.00), respectively. The independent predictors of depressive disorder included a family history of mental illness, chronic medical illnesses, unemployment, low educational status, divorced or widowed, poor social support, and current alcohol use or khat chewing. Single, males, divorced or widowed, and current consumers of alcohol were independent predictors for bipolar disorder.

**Conclusions and recommendation:**

The results of our investigation showed that bipolar illness and depression were significant public health issues. It was shown that although bipolar disorder is highly prevalent in the society, depression is a widespread concern. As a result, it is imperative that the relevant body grow and enhance the provision of mental health services. Furthermore, research on the effects and burdens of bipolar disorder in the community is required.

## Introduction

Worldwide, depressive disorder is widespread, and a leading cause of disability [1]. Depressive disorder is characterized by sadness, loss of interest or pleasure, feelings of guilt or low self-worth, disturbed sleep or appetite, feelings of tiredness, and poor concentration among these symptoms five or more symptoms persist for at least two weeks [2]. In its most severe form, depressive disorder can lead to suicide [3]. Whereas, bipolar disorder (BPD) is a mood disturbance Characterized by persistent and abnormally expansive, irritable, and elated mood; increased energy and goal-directed activity, decreased sleep, talkative, easily distractibility, and excessive risky activity, among these symptoms three or more symptoms persist or lasting for at least one- week duration [2].

Globally, Depressive disorder is rising at an alarming rate and more than 350 million people are affected by depressive disorder [4, 5]. More than 183.9 million disability-adjusted life years (DALY) are attributable to substance abuse and mental illness, according to statistics on the global burden of disease; depressive disorders accounting for more than 40.5% of these [6]. It is one of the most pressing public health problems associated with substantial poor quality of life, and interaction with others, comorbidity with other illnesses, impairment in cognition and emotion, and high mortality [7]. It is the leading cause of suicide. Depressive disorder leads individuals to health-related problems like suicide, diabetes, arthritis, and substance [5], and increases the mortality rate by four times compared to healthy individuals [7].

Costing more than 183.9 million is the report on the worldwide burden of disease linked to substance abuse and mental disorders. Beyond 7% of DALY cases have bipolar disorder [6]. In addition to the expense of treatment, bipolar disorder also raises the risk of unemployment, reduced productivity, and increased mortality [8]. Furthermore, risky sexual behavior, low quality of life, functional disability, suicide, and interpersonal interactions are all significantly impacted by bipolar disease [9–12].

Since BPD is a chronic condition and has an early onset, it accounts for more disability-adjusted life-years (DALYs) than neurological and other chronic medical (e.g. cancer) [13]. It significantly affects the emotions, mental health, self-care, and interpersonal relationships of the patient [14]. Because of the significant financial losses associated with receiving mental health services and patient care, it also places a heavy burden on the partner and their family [15].

According to a systematic review and meta-analysis of 90 publications, the combined lifetime and annual community prevalence of depressive disorder in the six continents and 30 countries studied was 10.8% and 7.32%, respectively [16]. In Ethiopia, the prevalence of depressive disorder was between 7.4–41% [17–20]. Risk factors of depressive disorder include the following: family history of a psychiatric disorder, history of mental illness, women, urban residence, widowed and divorced, chronic medical illness, immigrants, older age, ever and current substance use that is using once in lifetime and using of substance in the last three months (alcohol, khat, and tobacco) [21], unemployment, low educational status, poor social support, and history of neurological disorder like epilepsy [17, 22–27],

The prevalence of BPD varied from 0.1 to 25% globally [28, 29]. Bipolar disorder was 0.6% more common in men and 0.3% in women in Ethiopia over a lifetime [30]. Genetics, early life trauma, pregnancy complications, perinatal infections, childhood sexual abuse, cannabis and cocoaaine use, mental illness in the family history, and abnormality in thyroid hormone, being a man, having a high socioeconomic status, and doing well academically were found to be strongly correlated factors with bipolar illness [31–33].

In Africa, epidemiological evidence used for clinical, teaching, and research purposes was taken from the developed countries [34]. In Africa, there is poor awareness, poor perception, poor mental health service coverage, and few mental health professionals compared to developed countries [35, 36], Because of this depressive and bipolar disorders are underdiagnosed and underreported in Africa [37].

Even though depressive disorder and bipolar disorder cause a significant burden to the family and country, in Africa there is very limited epidemiological evidence, especially in bipolar disorder in the community setup [34]. So, this study has paramount significance in providing baseline data on the prevalence of depressive disorder and bipolar disorder. In addition, it can be a foundation for other studies and stakeholders to design early detection and intervention strategies. Therefore, this study aimed to assess the Prevalence of depressive disorder, bipolar disorder, and their associated factors among residents living in Kersa, Haramaya, and Harar Health and Demographic Surveillance Sites in Eastern Ethiopia.

## Methods and materials

### Study setting and period

Kersa HDSS was established in 2007, and Harar and Haramaya were added later, making the Hararghe Health and Demographic Surveillance Sites (HDSS) located in eastern Hararghe, Oromia Region. The HDSS consists of rural and urban kebeles. Most inhabitants are farmers, with a minority working in small-scale trade, government posts, or casual laborers [28]. The study was conducted from October 1 to 30, 2022.

### Study design

A community-based cross-sectional study design was employed.

### Population and eligibility

#### Source population

All residents living in Kersa, Haramaya, and Harar Health and Demographic Surveillance Sites (HDSS).

#### Study population

Residents who were selected by systematic random sampling from Kersa, Haramaya, and Harar Health and Demographic Surveillance Sites (HDSS) sites.

### Inclusion and exclusion criteria

#### Inclusion criteria

All adults aged 18 years and above who resided in the district for at least six months.

#### Exclusion criteria

Participants who were unable to communicate due to serious health illnesses.

### Sample size determination and sampling technique

Based on the particular goals, an estimate of the sample size was made. A single population proportion formula [p = (Zα/2) 2 p (1-p)/d2] was used to determine the sample size for objectives involving prevalence. Using the Double Population Proportion Formula and the Open Epi online software, the sample size was estimated for the associated factors’ objectives.

Accordingly, for the first objective, the sample size was estimated based on the assumption that the prevalence of depressive disorder was 17.5% [24], with a margin of error 5%, with a design effect of 1.5 (due to the multistage sampling of study participants) and 10% non-response rate; thus, 367 participants were considered as the minimum required sample size. The sample size for the second specific objective was estimated considering factors associated with substance use (male), the two-sided confidence level of 95%, 5% margin, 80% power, a design effect of 1.5, 10% non-response rate, and the ratio of exposed to unexposed 1:1; the sample size was 377. However, this study was part of a larger project entitled “Mental illness, substance use, and stigma in the community of Kersa, Haramaya, and Harar Health and Demographic Surveillance System, Eastern Ethiopia,” with a sample size of 1,416. Therefore, 1,416 was taken as the final sample size.

### Sampling technique and procedure

**Fig. 1:**
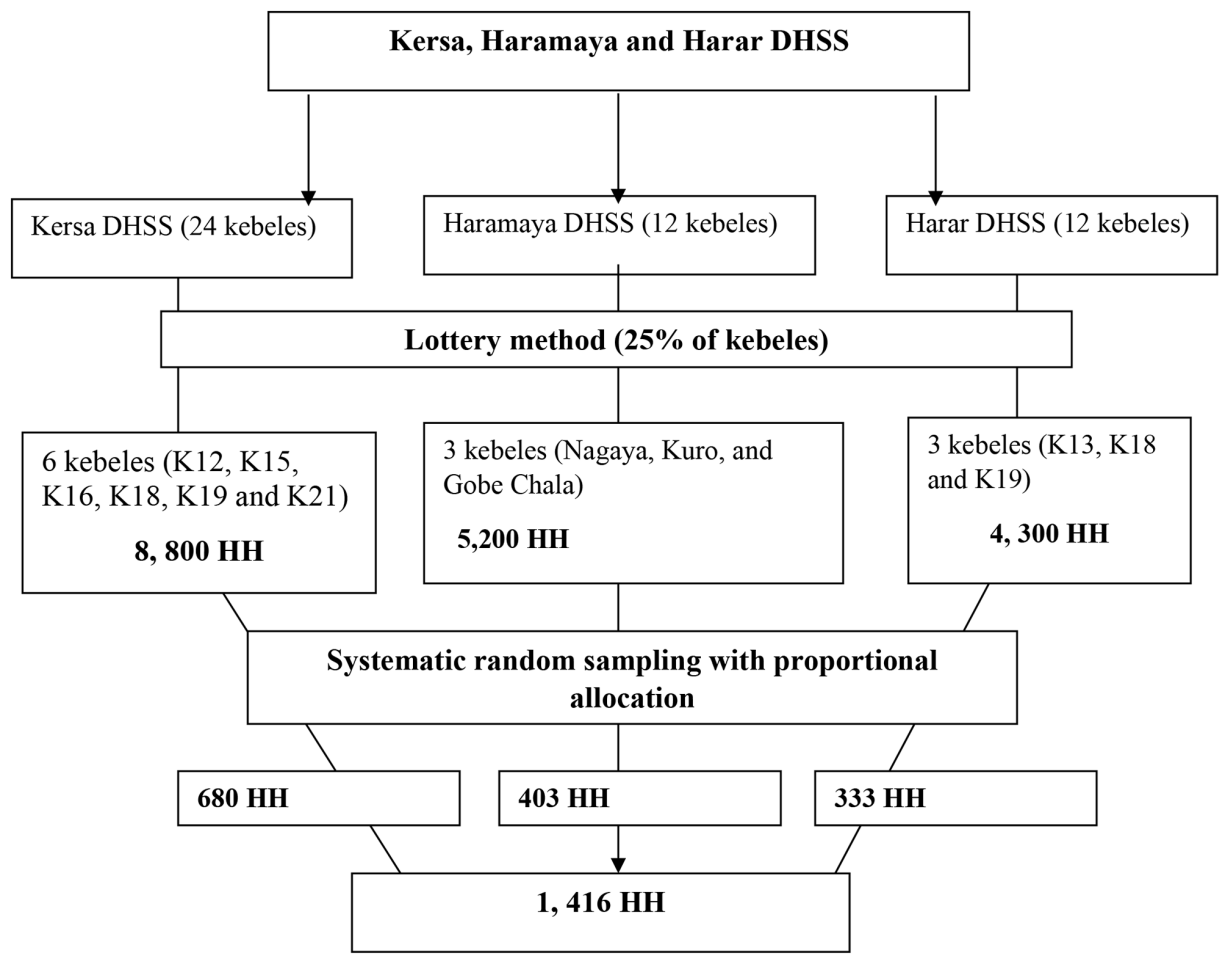
Schematic presentation of sampling procedure for assessing the depressive disorder and bipolar disorder in Kersa, Haramaya and Harar Health and Demographic Surveillance Sites, Eastern Ethiopia

A multi-stage probability sampling was used. There are 24 kebeles in Kersa and 12 in Haramaya and Harar DHSS. First, 25% of sample kebeles were selected by lottery from each site. Then, the total sample size was proportionally allocated to each kebele. Next, each household was selected by systematic random sampling. The first household was selected randomly; if there was more than one study participant (individuals aged 18 or above) in a home, a lottery method was used to limit one person to participate (Fig. 1).

### Data collection instruments and measurements

A structured questionnaire was used to collect all required data using a face-to-face interview. The questionnaire contained questions to assess the socio-demographic economics, depressive disorder., bipolar disorder, substance use, and social support of the participants.

#### Depressive disorder

the DSM-5 diagnostic criteria, which includes nine symptoms, was used to assess the major depressive disorder symptoms of the study participants [38].

#### Bipolar disorder

the DSM-5 diagnostic criteria for bipolar disorder was used. The tool contains seven questions that assess the symptoms of bipolar disorder [38].

#### Alcohol Smoking and substance involvement screening test (ASSIST)

The Alcohol, Smoking, and Substance Involvement Screening Test (ASSIST) assessed psychoactive substance use. This tool was developed by the World Health Organization with a sensitivity and specificity of 97 and 90, respectively. ASSIST is a brief screening questionnaire assessing current and lifetime psychoactive substance use [21, 38].

#### Social support assessed by the Oslo social support (Oslo-3)

The Oslo-3, referred to as the 14-point Social Support Scale, is a brief and economical instrument assessing the level of social support. The Oslo-3 consists of only three items inquiring about the number of close confidants, the sense of concern from other people, and the relationship with neighbors, focusing on the accessibility of practical help. Individuals who scored 12–14, 9–11, and 3–8 were considered to have strong, moderate, or poor social support, respectively [39, 40].

### Study variables

#### Dependent variables

- Depressive disorder.
- Bipolar disorder.

#### Independent variables

- **Socio-demographic and economic characteristics** (age, sex, religion, occupation, educational status, residence, average monthly income, living conditions).
- **Clinical factors** (history of mental illness, history of chronic medical illness, family history of mental illness, anxiety).
- **Psychosocial and behavioral factors**: social support.
- **Sbustance use(alcohol, Tobacco, khat use)**

### Data collectors

Nine health professionals with BSc holders collected the data, and three with an MSc supervised the data collection process.

### Data quality control

The structured questionnaire was first prepared in English and translated into Amharic and Afan Oromo; it was then re-translated to the English version by a language expert to check its consistency. In addition, we adapted standardized tools specific to the research objectives to ensure data quality. Finally, a pretest was done on 5% of the sample size one week before actual data collection in the kebeles not included in the sample to evaluate the acceptability and applicability of procedures and tools. To maintain the completeness and consistency of the questionnaires, the supervisors and investigators closely supervised the data collectors. In addition, the data collectors and the supervisor received two days of training.

### Data processing and analysis

After collection, data were cleaned checked for completeness, and coded. Data were entered into the computer using Epi-Data 3.1 and imported to SPSS version 26 for analysis. Descriptive statistical tests were used to provide a clear distribution of the data. Numerical variables were described as mean and standard deviations, while categorical variables were expressed as frequencies and percentages. Both binary and multivariate logistic regression were performed for analysis. For all models, variance inflation factors (VIF) tested the multi-collinearity of the independent variables. In addition, the model goodness of fit was checked, and a *p*-value of < 0.05 was considered the cut-off for statistical significance in the final analysis model.

### Ethical consideration

Ethical clearance was received from the Institutional Health Research Ethics Review Committee (IHRERC) of the College of Health and Medical Sciences at Haramaya University. All methods of the study performed according to the guideline and regulations of Helsinki. Next, data collection was initiated after a letter from the corporation was obtained from Haramaya University College of Health and Medical Sciences and given to each woreda and kebele administrator. Official permission was secured from the woreda and kebele administrators. Additionally, written voluntary informed consent was obtained from all participants after explaining the purpose and importance of the study. For all illiterate informed consent was obtained from the legal representative. Participation in the study was voluntary, and all the information collected from the participants was kept confidential under the researcher’s custody. Interviewers were trained to identify high-risk participants and those needing immediate counseling and refer them to a psychologist or psychiatrist.

## Result

### Socio-demographic characteristics of the participants

A total of 1,411 study participants were enrolled in the survey. More than half, 736 (52.2%), were female. The mean age was 34.11 (± SD, 11.226). The majority, 833 (59.0%), of participants were married. Of the participants, 427 (30.3%) had an educational status of diploma and above, but 492, 34.9%, could not read or write. Concerning occupation, 45.2% were employed (government or self), whereas 32.5% were farmers or housewives. The majority, 65.2% of participants were from rural areas (Table 1).

**Table 1:** Socio-demographic Characteristics of Study Participants in the Community of Kersa, Haramaya, and Harar Health and Demographic Surveillance System, Eastern Ethiopia, 2022 (N = 1411)

Variables		Frequency	Percentage
Sex	Male	675	47.8
	Female	736	52.2
Age	< 30	622	44.1
	31–40	502	35.6
	41–50	166	11.8
	≥ 51	121	8.6
Marital status	Single	294	20.8
	Married	833	59.0
	Divorced/ Widowed	284	20.1
Educational status	Unable to read and write	492	34.9
	1-8th grade	204	14.5
	High school (9-12th grade)	288	20.4
	Diploma and above	427	30.3
Occupational status	Employed	638	45.2
	Farmer/housewife	458	32.5
	Unemployed	315	22.3
Residence	Rural	920	65.2
	Urban	491	34.8
Living circumstance	With families	1302	92.3
	Alone	109	7.7

### Factors related to clinical, social support, and substance use characteristics of participants

Of the total study participants, 124 (8.8%) had a history of mental illness. Furthermore, 188 (13.3%) had a family history of mental illness. Almost 13% of the participants had a history of chronic illnesses like diabetes mellitus or hypertension. About three out of every five study participants, 59.0%, had used substances (i.e., khat, alcohol, or cigarette). Khat was the most commonly used substance, 783 (55.5%), followed by cigarettes and alcohol, 274 (19.4%) and 156 (11.1%), respectively. More than half, 56.4%, of our study participants, were current substance users (i.e., khat, alcohol, or cigarettes). The majority, 746 (53.9%), chewed khat in the past three months, whereas 255 (18.1%) and 125 (8.9%) smoked cigarettes and used alcohol, respectively. Only 15% and less than half, 46.1%, of the study participants had strong and moderate social support (Table 2).

**Table 2:** Clinical, social support, and substance use characteristics of study participants in the Community of Kersa, Haramaya, and Harar Health and Demographic Surveillance System, Eastern Ethiopia, 2022 (N = 1411)

Variable		Frequency	Percentage	
Presence of a history of mental illness	Yes	124	8.8	
	No	1287	91.2	
Family history of mental illness	Yes	188	13.3	
	No	1223	86.7	
Having a history of chronic medical condition	Yes	181	12.8	
	No	1230	87.2	
Ever use of substances (alcohol, cigarette, khat, …)	Yes	832	59.0	
	No	579	41.0	
Current use of substances (alcohol, cigarette, khat, …)	Yes	796	56.4	
	No	625	42.6	
Level of social support	Poor	548	38.8	
	Moderate	651	46.1	
	Strong	212	15.0	

### Prevalence of depressive disorder

The current prevalence of depressive disorder among our study participants was 6.7% (at 95% CI: 5.40,-8.20). Out of the participants with depressive disorder, nearly one-third were 35–39 years old, and the majority (57.4%) had poor social support. In addition, over 80% of depressed individuals had lifetime exposure to using a substance, and three-fourths of them were using a substance within the preceding three months.

### Factors associated with depressive disorder

After analysis using the binary logistic regression model, the following were associated with depressive disorder with *p*-values of less than 0.25 were entered into a multivariable logistic regression model: sex, age, marital status, educational status, residency, history of mental illness, occupational status, family history of mental illness, current use of a substance, and social support.

However, in the last analysis model, residency, positive family history of mental illness, history of chronic medical illness, unemployment, low educational status, divorced or widowed, having poor social support, and current alcohol or khat user were found to be independent predictors for depression. Hence, participants living in an urban area had over four times increased odds of having significant depressive-like symptoms compared to their rural dwelling counterparts (AOR = 4.253, 95% CI: (1.999, 9.048). Study participants with a family history of mental illness had twice the risk for depressive disorder than those with no family history (AOR = 2.240, 95% CI: (1.201, 4.178).

Our study results showed that a history of chronic medical illness raised the risk of having depressive disorder by 3.73 (AOR = 3.727, 95% CI: (2.047, 6.787). The unemployed participants had over a 2.7 times higher risk for depressive disorder compared to the self-employed participants or government employees (AOR = 2.732, 95% CI: (1.399, 5.336). Study participants with the lowest educational status had over a 4.4 times higher risk for depressive disorder compared to those who had earned a diploma or above (AOR = 4.437, 95% CI: (2.268, 8.680). Divorced study participants were two times more likely to have depressive disorder than their married counterparts (AOR = 2.283, 95% CI: (1.150, 4.532). Poor social support increased study participants’ risk for depressive disorder by four times compared to those with strong social support (AOR = 4.069, 95% CI: (1.355, 12.211). Our study participants who currently used alcohol or khat had four- and two-times increased risk of having depressive disorder compared to their abstained counterparts (AOR = 4.050, 95% CI: (2.060, 7.961) and (AOR = 2.009, 95% CI: (1.091, 3.701), respectively (Table 3).

**Table 3:** Factors associated with depressive disorder in the Community of Kersa, Haramaya, and Harar Health and Demographic Surveillance System, Eastern Ethiopia, 2022 (N = 1411)

Variables		Depression		COR (95%CI)	AOR (95%CI)	*P*-Value
		Yes	No			
Residency	Rural	82	838	3.91(2.11, 7.23)	**4.25(1.99, 9.05)**	**< 0.001**
	Urban	12	479	1	**1**	**1**
Family history of illness?	Yes	32	156	3.84(2.43, 6.07)	2.24(1.20, 4.18)	0.011
	No	62	1317	1	1	
Having a history of chronic medical illness?	Yes	31	150	3.83(2.41, 6.08)	**3.73(2.03, 6.79)**	< 0.001
	No	63	1167	1	1	
Occupation	Employed	45	593	1	1	
	Farmer/housewife	15	443	0.45(0.25, 0.81)	0.84(0.37, 1.89)	0.676
	Unemployed	34	281	1.59(0.99, 2.54)	**2.73(1.39, 5.34)**	**0.003**
Educational status	Unable to read and write	46	446	1.89(1.12, 3.21)	**4.44(2.27, 8.68)**	**< 0.001**
	1-8th grade	16	188	1.57(0.80, 3.05)	2.102(0.97, 4.58)	0.062
	9th -12th grade	10	278	0.66(3.9, 1.42)	0.78(0.27, 1.69)	0.126
	Diploma and above	22	405	1	1	1
Marital status	Single	20	274		1.48(0.64, 3.25)	0.371
	Divorced/widowed	35	249		**2.28(1.15, 4.53)**	**0.018**
	Married	39	794	1	1	1
Age	≤ 30			1.49(0.85, 2.59)	1.01(0.39, 2.58)	0.982
	31–40			2.86(1.77, 4.62)	1.56(0.65, 3.77)	0.317
	41–50			0.05(0.63, 1.24)	1.40(0.53, 3.73)	0.498
	≥ 51			1	1	1
Social support	Poor	54	494	5.68(2.03, 15.89)	**4.07(1.36, 12.21)**	**0.012**
	Moderate	36	615	3.049(1.07, 8.65)	1.85(0.59, 5.82)	0.296
	Strong	4	208	1	1	1
Current use of alcohol	Yes	24	101	4.13(2.49, 6.85)	**4.05(2.06, 7.96)**	**> 0.001**
	No	70	1216	1	1	1
Current use of khat	Yes	68	678	2.47 (1.55, 3.92)	**2.01(1.09, 3.70)**	0.025
	No	26	639	1	1	1

### Prevalence of bipolar disorder

The prevalence of bipolar disorders (i.e., moderate to high risk) was 2.1% (n = 30). Out of those, 27(76.7%) were males and 7(23.3%) were females. Half of those with bipolar disorder were single, 53.3% could not read and write, and the majority (86.7%) were from rural areas. 73.3% of those with bipolar had used a substance at least once, and 70.0% were current users. Three out of four participants with moderate to high risk of bipolar disorder had poor social support.

### Factors associated with bipolar disorder

In the bivariate logistic regression model, sex, residence, marital status, and current khat use were associated with bipolar disorder at a *p*-value less than 0.25. However, in the final analysis, males, single, divorced or widowed, and current alcohol use were found to be independent predictors for bipolar disorder. Hence, male study participants had close to triple times increased risk for bipolar disorder than their female counterparts (AOR = 2.818, 95% CI: (1.140, 6.965). Furthermore, our study results showed that single participants had more than seven times increased odds of having bipolar disorder (AOR = 7.437, 95% CI: 7.437 (2.651, 20.863). Those who are divorced or widowed had more than five times increased risk when compared to their counterparts (AOR = 5.559, 95% CI: (1.863, 16.590) (Table 4).

**Table 4:** Factors associated with Bipolar Disorder in the Community of Kersa, Haramaya, and Harar Health and Demographic Surveillance System, Eastern Ethiopia, 2022 (N = 1411)

Variables		Symptoms of Bipolar disorder		COR (95%CI)	AOR (95%CI)	*P*-Value
		Yes	No			
Sex	Male	23	652	3.67(1.57, 8.62)	**2.82(1.14, 6.97)**	**0.025**
	Female	7	729	1	1	1
Marital status	Single	15	279	8.90(3.21, 24.72)	**7.437(2.65, 20.86)**	**< 0.001**
	Divorced/widowed	10	274	6.04(2.05, 17.84)	**5.56(1.86, 16.59)**	**0.002**
	Married	5	828	1	1	1
Residency	Rural	26	894	1	1	1
	Urban	4	487	0.20(0.09, 0.81)	0.42(0.14, 1.24)	0.118
Current use of khat	Yes	21	725	5.50(2.52, 12.04)	1.79(0.77, 4.15)	0.178
	No	9	656	1	1	**1**

## Discussion

Our study aimed to investigate the prevalence and associated factors of depressive disorder and bipolar disorder. The prevalence of depressive and bipolar disorder was 6.7% at 95% CI (5.4–8.2) and 2.1% at 95% CI (1.4–3.0), respectively. In addition, the following were associated with depressive disorder, unemployment, divorced or widowed, low educational status, urban residence, family history of mental illness, chronic medical illness, poor social support, and current alcohol or khat use. At the same time, being single and divorced or widowed was associated with bipolar disorder.

The prevalence of depressive disorder in our study area was 6.7%. Our study results were comparable to the study done in Nigeria, which was a comparative community-based study among the rural and urban communities and the overall prevalence of depressive disorder was 5.2% by using the general health questionnaire (GHQ 12) as a screening tool [41] and our study results are lower than from a community-based cross-sectional study done among adults conducted in Ebinat town and South Wolo in Northern part of Ethiopia the by using PHQ − 9 and Center for Epidemiologic Studies Depression scale (CES-D) to assess the depression, the prevalence of depression showed that 17.5% [24] and 51.0% [42] respectively. The finding was also lower than the Malaysian study which was a community-based cross-sectional study that revealed the prevalence of depressive disorder at 10.3% using a PHQ-9 cut-off point of 10 and above [43]. The South Wolo study, which took place following the liberation of the invasion by the TPLF-led force, may provide an explanation for the prevalence variation. The target population includes residents who live in the community and seek refuge, which may exacerbate depressive symptoms. The Malaysian study found that depressive disorder affects women more frequently than men due to hormonal differences, the impact of childbirth, the presence of distinct psychosocial stressors, and behavioral models of learned helplessness. Another possible explanation is that the instruments used to assess depression differ, and the majority of participants were women [44].

On the opposite side, the finding of this study was higher than a study conducted in Canada, 4.8% which was a descriptive epidemiological study among adults in the community setting [45], The discrepancy from Canada’s study might be the lower figures for Canada may reflect the lower rate of poverty in Canada (17%) than in low- and middle-income countries (28%) [46, 47]. According to the social causation of mental illness being poor in the economy or decline in daily income, and struggling to secure food, household, and shelter will lead to depressive disorder [48, 49]. The disparity may arise from the fact that the study was carried out in developing nations and the participants were people living in low- and middle-income countries. In this study, where women are more likely to experience depression, over half of the participants were women. In previous research, over half of the participants were men. The impact of childbirth, behavioral models of learned helplessness, variations in psychosocial stressors, and hormone differences are among the theories put forth to explain why women are more likely than men to develop depressive disorders [44].

The odds of having depressive disorder were more than two times more likely to occur among divorced/widowed individuals than married. This finding agrees with previous study [50]. This could mean that being widowed or divorced is viewed as a major loss and source of stress. A divorce or death may also result in an increased family burden, encompassing everyday and financial responsibilities.

According to these study findings, unemployment was associated with depressive disorder, which aligns with previous studies in Ethiopia [17]. Since employment is one of the main factors influencing both mental and physical health as well as economic standing, unemployment has a major negative impact on poverty Moreover, unemployment is regarded as a major stressor that exacerbates depression and results in changes to social and familial roles [51–53]. Poverty and mental disorders can coexist in a vicious cycle as mental illness exacerbates the financial circumstances of those it affects.

Regarding social support, depressive disorders are higher among individuals with poor social support, as in previous Ethiopian studies [54, 55]. Social support is one of the determinant of health that refers to the social integration, bonds, and relationships that exist between the group. Supportive interpersonal relationships, and bonds between them help to have good mental health [56]. In addition to this social support can minimize or suppress the effect of stress that predisposes to depression. From another perspective, the domain of social support includes social networks, bonds, and social contacts with others, so from the nature of depression, they may isolate from social networks, and social contacts since the study design doesn’t tell cause and effect. This study also shows that those living in rural settings are more likely to develop depressive disorder than those in urban settings. The implication for those might be there may be strong social support and better way of life in urban than in rural.

Additionally, this study indicated that having a family history of mental illness was one of the predisposing factors for depressive disorder. This is concurrent with a study from Ethiopia [55]. It is known that generally mental illness runs within the family [57]. Research’s reported that there is heritability among first-degree relatives of more than 40–50% and more than two times the risk to have depressive disorder than counterparts [58]. In addition to this evidence reported that persons with positive family history of mental illness are vulnerable and may require little provocation to have another episode of depressive disorder [59]. Another reason might be the burden of caring for mentally ill patients is stress which causes a load and a burden on the family, again which causes depressive disorder.

Furthermore, current khat use was an independent predictor of depression, which agrees with previous studies in Ethiopia [60, 61]. The possibility might be that a depressed person might use khat as self-medication. Another finding of this study revealed that having a chronic medical illness was a risk factor for depressive disorder. This agrees with the findings of several studies [19, 62]. Evidence suggests that there is a bidirectional association between Chronic medical illness and mental illness [63]. For example, medical illness predisposes and precipitates depressive disorder by creating major physiological, psychological, and emotional disturbances and financial stressors. Another reason might be chronic medical illness can cause significant changes, such as disability, dependency, and decreased self–esteem and self-confidence, resulting in depressive disorder [22, 43].

The current study also showed that participants with low educational status were more likely to have depressive disorder than their counterparts. The finding was consistent with the study [64]. The implication for this might be education may enhance coping mechanisms for stressful life events. It will also increase decision-making abilities and problem-solving abilities. Current alcohol users were more likely to have depressive disorder than non-users. This finding is similar to a previous study [42, 65]. Alcohol decreases the concentration of serotonin in the blood, which is a predisposing factor for depressive disorder [66].

This study revealed that the Prevalence of bipolar disorder was 2.1%, which is in line with studies from Ethiopia that found 1.83% [34] and in Italy 3% [67]. However, the finding is lower than the previous study from the USA, 3.7% [68]. This discrepancy might be because of the tool difference in assessing bipolar disorders. The USA study used the mood disorder questionnaire (MDQ), whereas this study used DSM-5 diagnosing criteria.

Being single and divorced or widowed was also associated with bipolar disorder. This finding is congruent with the findings in Australia [69]. The implication might be that being divorced or widowed leads to a negative adverse life event and significant losses, creating stress and mental illness. However, since the onset of bipolar disorder is often at an early age and before marriage, and this is a cross-sectional study, it is difficult to ascertain the direction of causality.

The odds of having bipolar disorder were nearly three times occur among men than women. The possible implication for this might be manic episodes of bipolar disorder more occur among men than women and depressive episodes and rapid cycling among women [70]. Since this study used DSM-5 diagnosing criteria to assess bipolar disorder, we are not able to assess depressive episodes of bipolar disorder.

### Limitations and strengths of the study

With a large enough sample size, this study showed the prevalence of depressive and bipolar disorders is common in the Eastern Ethiopian community. Nevertheless, we had no control over the temporal relationship between the risk factor and the outcome variable because this was a cross-sectional study. Another drawback is that the study was unable to identify participants’ bipolar disorder with depressive episodes because we evaluated both depressive and bipolar-like symptoms.

## Conclusion and recommendation

Overall, the prevalence of depression and bipolar disorder was similar to that of previous studies conducted in Africa. Both bipolar disorder and depressive disorder are significantly influenced by sociodemographic factors. Depressive disorder was also significantly influenced by social support, alcohol consumption at the time, and a family history of mental illness. Therefore, the present data may be used as a starting point, at both practice and policy-making levels, for expanding and improving the quality of mental health service delivery. Furthermore, more research on the costs and effects of bipolar disorder in the community is required, as well as community awareness campaigns from the relevant body is needed.” The effectiveness of mental health services could then be measured through research. It is necessary to evaluate bipolar disorder independently or in combination to identify bipolar disorder with depressive episodes.

## Data Availability

The datasets used and/or analyzed during the current study are available from the corresponding author upon reasonable request.
